# Mesenchymal stem cells promote pancreatic β-cell regeneration through downregulation of FoxO1 pathway

**DOI:** 10.1186/s13287-020-02007-9

**Published:** 2020-11-25

**Authors:** Rahul Khatri, Sybille Mazurek, Sebastian Friedrich Petry, Thomas Linn

**Affiliations:** 1 Third Medical Department, Clinical Research Lab, Justus Liebig University Giessen, Giessen, Germany; 2 Institute of Veterinary Physiology and Biochemistry, Justus Liebig University Giessen, Giessen, Germany; 3 Clinical Research Unit, Centre of Internal Medicine, Friedrichstrasse. 20/ Aulweg 123, 35392 Giessen, Germany

**Keywords:** Mesenchymal stem cells, Intrapancreatic transplantation, Partial pancreatectomy, Epidermal growth factor and pancreatic β-cell proliferation

## Abstract

**Background:**

Mesenchymal stem cells (MSC) are non-haematopoietic, fibroblast-like multipotent stromal cells. In the injured pancreas, these cells are assumed to secrete growth factors and immunomodulatory molecules, which facilitate the regeneration of pre-existing β-cells. However, when MSC are delivered intravenously, their majority is entrapped in the lungs and does not reach the pancreas. Therefore, the aim of this investigation was to compare the regenerative support of hTERT-MSC (human telomerase reverse transcriptase mesenchymal stem cells) via intrapancreatic (IPR) and intravenous route (IVR).

**Methods:**

hTERT-MSC were administered by IPR and IVR to 50% pancreatectomized NMRI nude mice. After eight days, blood glucose level, body weight, and residual pancreatic weight were measured. Proliferating pancreatic β-cells were labelled and identified with bromodeoxyuridine (BrdU) in vivo. The number of residual islets and the frequency of proliferating β-cells were compared in different groups with sequential pancreatic sections. The pancreatic insulin content was evaluated by enzyme-linked immunosorbent assay (ELISA) and the presence of hTERT-MSC with human Alu sequence. Murine gene expression of growth factors, β-cell specific molecules and proinflammatory cytokines were inspected by real-time polymerase chain reaction (RT-PCR) and Western blot.

**Results:**

This study evaluated the regenerative potential of the murine pancreas post-hTERT-MSC administration through the intrapancreatic (IPR) and intravenous route (IVR). Both routes of hTERT-MSC transplantation (IVR and IPR) increased the incorporation of BrdU by pancreatic β-cells compared to control. MSC induced epidermal growth factor (EGF) expression and inhibited proinflammatory cytokines (IFN-γ and TNF-α). FOXA2 and PDX-1 characteristics for pancreatic progenitor cells were activated via AKT/ PDX-1/ FoxO1 signalling pathway.

**Conclusion:**

The infusion of hTERT-MSC after partial pancreatectomy (Px) through the IVR and IPR facilitated the proliferation of autochthonous pancreatic β-cells and provided evidence for a regenerative influence of MSC on the endocrine pancreas. Moderate benefit of IPR over IVR was observed which could be a new treatment option for preventing diabetes mellitus after pancreas surgery.

**Supplementary information:**

The online version contains supplementary material available at at 10.1186/s13287-020-02007-9.

## Introduction

Distal pancreatectomy is a common surgical procedure employed when tumours or pseudocysts occur in the tail of the pancreas. However, while it is not a common side effect of the intervention, several risk factors for the occurrence of a pancreoprivic diabetes mellitus (type 3c) have been identified, i.e. age, obesity and chronic pancreatitis [1, 2]. Of note, the glucagon-secreting alpha-cells and pancreatic polypeptide-secreting PP-cells are mainly localized in the head and tail of the pancreas [3]. Therefore, the entire endocrine function of the organ is abrogated in type 3c diabetes mellitus, while type 1 and 2 diabetes both are characterized by an impaired production and efficacy of insulin, respectively. The severity of the resulting metabolic disorder is naturally highly dependent on the extent of the resection. However, in daily practice, patients often suffer from a hard to manage so-called brittle diabetes mellitus: since the deficiency of glucagon markedly impairs the hepatic gluconeogenesis, severe hypoglycaemia can occur frequently and unexpectedly upon treatment with exogenous insulin [4]. Therefore, means to preserve the endocrine function and facilitate an insulin-free treatment after pancreatectomy might potentially improve the procedure’s overall long-term outcome.

In this regard, multipotent yet endogenous mesenchymal stem cells/stromal cells might facilitate novel regenerative therapies. These cells have a fibroblast-like structure and self-renewal property. They were reported to differentiate into chondrocytes, cardiomyocytes, adipocytes, myoblasts and pancreatic-like cells in vitro [5–8]. MSC can be derived from a wide variety of tissues such as bone marrow, adipose tissue, dental pulp, Wharton’s jelly, umbilical cord matrix blood or placenta with ease and can be rapidly expanded in bioreactors ready for treatment in patients [9]. Moreover, they have immunomodulatory properties and release growth factors at injury sites [10–12]. Indeed, MSC administration showed potential to reverse hyperglycaemia in experimental models but the mechanism is still elusive [13–16].

To compensate for β-cell deficiency, the expansion of the residual cell proliferation, reduction of cell death events or both are required. There is evidence for a regenerative potential of the adult pancreas, i.e. it was proposed that partial pancreatectomy (Px) induces β-cell proliferation derived from pre-existing cells with minimum neogenesis [17]. Research focused on replenishing pancreatic β-cells either by initiating division of residual or conversion to β-cells from other pancreatic cell types [18–23]. Human bone marrow MSC were transduced with a retroviral vector including human telomerase (hTERT) gene to create an immortalized bone marrow-derived human telomerase reverse transcriptase mesenchymal stem cells (hTERT-MSC) line [24]. While primary MSC displayed a finite proliferative capacity, the introduction of the hTERT gene was reported to provide infinite in-vitro expansion [24, 25]. hTERT-MSC showed differentiation potential towards adipogenic, osteogenic and neural lineages [26]. Moreover, its safety was recently demonstrated in a skin tumour NMRI nu/nu mice model [27]. Therefore, we utilized partially pancreatectomized NMRI nude mice to investigate the regenerative potential of hTERT-MSC. The majority of MSC supplied via a systemic route are entrapped in the microvasculature of the lung, diminishing the beneficial effect on the pancreas [28, 29]. Therefore, we compared the potency of hTERT-MSC by local (intrapancreatic route (IPR)) or systemic injection (intravenous route (IVR)). We provide substantial evidence of MSC’s influence on the proliferation of pancreatic β-cells as well as on activation of AKT/PDX-1/FoxO1 signalling cascade in pancreatic β-cells.

## Methods

### Animals

**Fig. 1:**
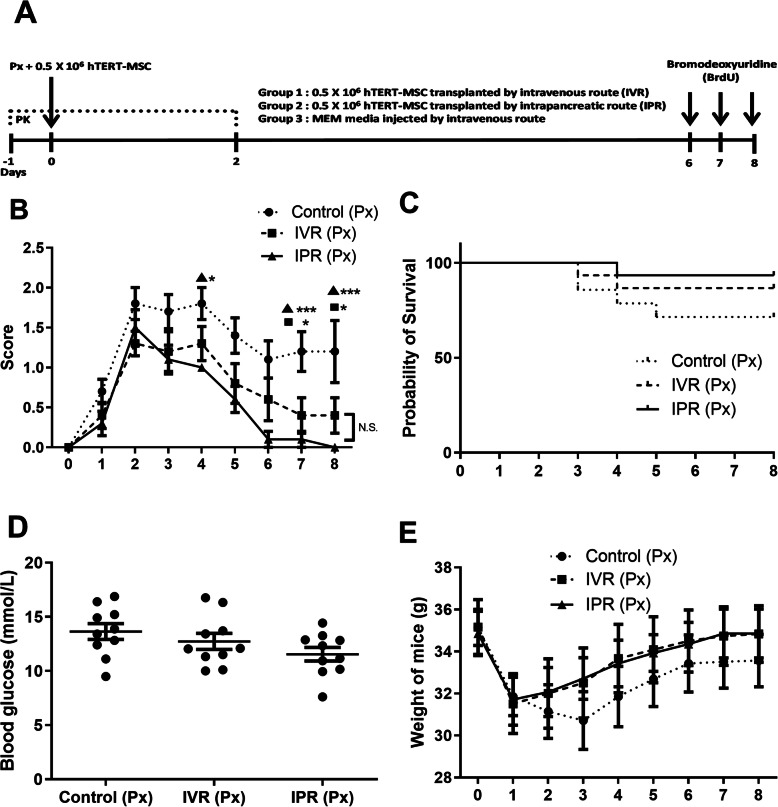
Experimental design, glucose levels and body weight. **a** Diagrammatic representation of an experimental scheme. Pain killer (PK) tramadol (0.2 per mg mouse a day) was administered in drinking water for four consecutive days (− 1, 0, 1 and 2 day). At 0-day mice were anaesthetized and Px was performed. Once Px was completed, 0.5 × 10^6^ hTERT-MSC were transplanted by intravenous route (IVR) or intrapancreatic route (IPR) on 0 days. Controls were pancreatectomized and injected with cell culture medium intravenously, *n* = 10. **b** Mice were evaluated daily for well-being (scoring sheet, refer supplement 1). IPR group revived faster from pancreatic insult than others, *n* = 10. **c** Percentage survival of mice following Px was similar in the three groups during the observation window of 8 days, *n* = 14. **d** Blood glucose levels were measured 8 days after Px and showed the lowest concentrations in IPR compared to other groups without significance, *n* = 10. **e** Body weights were measured daily, *n* = 10. Profiles were similar in the three Px groups and showed recovery to initial body weight after 8 days in the experimental groups, but not in the control group. Human telomerase reverse transcriptase mesenchymal stem cells (hTERT-MSC). Data are given as mean ± SEM, *p < 0.05, **p < 0.01, ***p < 0.001

Eight to 12-week-old male NMRI nude mice were purchased from Janvier (France). They were housed with a 12:12 h cycle (light:dark) at an appropriate temperature (24 ± 2 °C) and humidity (55 ± 10%). The mice were fed with the laboratory standard water and food (1324 TPF, ad libitum, Altromin, Germany). The experimental procedures were approved by the ethical committee according to the German Animal Welfare Law and Guidelines under the code 31/2017. The experimental design is illustrated in Fig. 1a.

### Culture condition and transplantation of hTERT-MSC

hTERT-MSC is a bone marrow-derived immortalized cell line produced by transfection with a retrovirus containing the hTERT gene [24, 30]. hTERT-MSC were seeded at a density of 5 × 10^4^ cells/cm^2^ in MEM (Minimum Essential Medium; Gibco, Germany) with 10% FBS (Biowest, USA), 1% L-glutamine (Invitrogen, Germany), 1% penicillin-streptomycin (Invitrogen, Germany) and incubated at 37 °C with 5% CO_2_ in the incubator. Cells were harvested after 70 to 80% confluency and detached with 5 ml trypsin-EDTA from T-175 culture flask. Enzymatic reaction was stopped with MEM (10% FCS). Cells were washed with PBS twice and resuspended in 5 ml. Cells were counted with trypan blue (dilution of 1:20; 5 μl hTERT-MSC and 95 μl trypan blue) in a Neubauer chamber. 5 × 10^6^ cells were resuspended in a 1 ml MEM media (without supplements) and from this stock 100 μl containing 0.5 × 10^6^ were considered for transplantation. Three distinct groups were established: control (received MEM through intravenous injection after pancreatectomy), IVR (transplanted 0.5 × 10^6^/100 μl hTERT-MSC through intravenous injection after pancreatectomy) and IPR (transplanted 0.5 × 10^6^/100 μl hTERT-MSC directly into the residual pancreas after pancreatectomy).

### Partial pancreatectomy (Px)

The mice were anaesthetized with ketamine (100 mg/kg body weight; Medistar, Germany) and xylazine (20 mg/kg body weight; Ceva, Germany) by an intraperitoneal injection. The surgical procedure was performed modifying the description of Migliorini and Preto (1970) [31]. The stomach and spleen were drawn out of the abdominal cavity through a midline incision and the transverse colon was pulled down to expose the junction of the superior mesenteric and splenic vein forming the portal vein. Then, the gastric and splenic pancreatic tissue were separated with fine-tipped forceps. A dissection was carried upward from the superior mesenteric vein to the spleen with the aid of cotton swabs and scissors. After the splenic vein was tied up with synthetic absorbable suture (Vicryl suture 5-0, Ethicon, USA), the gastrosplenic ligament was cut separating the spleen from the stomach. The mass of the resected portion of the pancreas was about 0.090 g, representing 50% of total pancreatic tissue (hemipancreatectomy) and leaving the duodenal and parabiliary portion of the pancreas.

In the IPR group, 0.5 × 10^6^/100 μl hTERT-MSC were filled in a 1-ml syringe (Braun, Germany) and injected slowly within 10 s in the deep parenchyma (3–5 mm) of the residual pancreas with a 26-gauge steel needle (0.45 × 13 mm; BD, USA), resulting in a visible depot. The needle was withdrawn slowly, and the injection site was observed for another 15 s to detect bleeding or leakage of the injected cells. The abdominal wall and upper skin were thereafter closed with synthetic absorbable suture. All animals were sacrificed on day eight.

### Pain management

From starting 1 day before hemipancreatectomy until two post-operative days, mice were treated with tramadol (2.5 mg = 0.25 ml/100 ml) in their drinking water. Additionally, meloxicam (1 mg/kg) was also administered subcutaneously on the day of the experiment and continued for another two successive days for better post-operative pain management.

### Quantification of insulin content in the residual pancreas

The residual pancreas was mechanically homogenized and dissolved in acid ethanol as documented previously [32, 33]. The supernatant was centrifuged (3000 rpm for 10 min at 4 °C) and its insulin content was measured employing a mouse Insulin ELISA kit (DRG Instruments GmbH, Marburg, Germany).

### Blood glucose measurement and BrdU incorporation

Blood glucose was measured with a hand-held glucose meter (One Touch® Ultra®2, LifeScan) by puncturing the tail vein after 8 days of hemipancreatectomy. The incorporation of Bromodeoxyuridine (BrdU) into the pancreatic cells was accomplished by injecting 100 mg BrdU/kg body weight in three successive days before sacrificing the mice.

### mRNA expression of growth factors, immunomodulatory molecules and specific β-cell development transcriptional factor

Total RNA was extracted from the pancreas by the peqGOLD TrifastTM reagent (peqlab, Germany). The RNA concentration was quantified by using a NanoDrop spectrophotometer (NanoDrop, USA) and 1 μg RNA was transcribed with the SuperScript III Reverse Transcriptase kit (Invitrogen, Germany). Real-time PCR (StepOnePlus, Applied Biosystems) was carried out with SYBR Green Supermix (Bio-Rad Laboratory, Germany), primers (primer concentration; 20 pM) and cDNA template. Cycling condition of each PCR reaction was initial denaturation at 95 °C for 10 min, followed by 40 cycles of denaturation (95 °C, 15 s), annealing (60 °C, 30 min) and extension (60 °C, 1 min), followed by the melting curve. Relative quantification of each gene was performed by ΔΔCT value method. Refer Table 1 for primer list.

**Table 1:** Primer list

Primer	Forward primer	Reverse primer
**Mouse EGF**	5′-TCTCGGATTGACCCAGAT-3′	5′-CCCAGACACCTTCCTCTCT-3′
**Mouse GLUT-2**	5′-TGTGCTGCTGGATAAATTCGCCTG-3′	5′-AACCATGAACCAAGGGATTGGACC-3′
**Mouse PDX-1**	5′-GAACCCGAGGAAAACAAGAGG-3′	5′-GTTCAACATCACTGCCAGCTC-3′
**Mouse Ins1**	5′-TATAAAGCTGGTGGGCATCC-3′	5′-GGGACCACAAAGATGCTGTT-3′
**Mouse Ins2**	5′-GGCTTCTTCTACACACCCATGT-3′	5′-AAGGTCTGAAGGTCACCTGCTC-3′
**Mouse FOXA2**	5′-GACATACCGACGCAGCTACA-3′	5′-TAGATCTCGCTCAGCGTCAG-3′
**Mouse PI3K**	5′-CTCTCCTGTGCTGGCTACTGT-3′	5′-GCTCTCGGTTGATTCCAAACT-3′
**Mouse AKT**	5′-ATCCCCTCAACAACTTCTCAGT-3′	5′-CTTCCGTCCACTCTTCTCTTTC-3′
**Mouse FoxO1**	5′-TTCAATTCGCCACAATCTGTCC-3′	5′-GGGTGATTTTCCGCTCTTGC-3′
**Mouse TNF-α**	5′-CATCTTCTCAAAATTCGAGTGACAA-3′	5′-TGGGAGTAGACAAGGTACAACCC-3′
**Mouse IFN-γ**	5′-CGGCACAGTCATTGAAAGCC-3	5′-TGCATCCTTTTTCGCCTTGC-3′
**Mouse RPL32**	5′-GGAGAAGGTTCAAGGGCCAG-3′	5′-GCGTTGGG ATTGGTGACTCT-3′
**Human Alu sequence**	5′-CATGGTGAAACCCCGTCTCTA-3′	5′-GCCTCAGCCTCCCGAGTA G-3′

### Immunohistochemistry

Immunohistochemistry was used to detect insulin, BrdU-labelled positive cells and FoxO1. Pancreatic tissues were fixed with 4% paraformaldehyde for 4 to 6 h and embedded in paraffin. Afterwards, 7 μm pancreatic tissue sections were cut and deparaffinized in citrate buffer for antigen retrieval. Sections were washed with 0.1% TRIS buffer, pH 7.6 and blocked with 1% goat serum (Biowest, France) for 20 min, followed by staining with the respective primary antibody (polyclonal guinea pig anti-insulin, DAKO, Germany and rabbit Anti-FOXO1A, Abcam, Germany) and kept overnight at 4 °C. Prior to the administration of the primary antibody, sections were treated with NaOH (1:11 diluted with distilled water), followed by washing with TRIS buffer. On the next day, the sections were washed with TRIS buffer and stained for the respective secondary antibody (alkaline phosphate conjugated affinity purified anti-guinea pig, Rockland, Germany, and alkaline phosphate conjugated affinity purified anti-rabbit, Rockland, Germany) with 5% mouse serum (Biowest, France) for 1 h at RT. Sections were washed with TRIS buffer. FoxO1 was developed with a reddish colour using a Fuchsin kit (Dako, Germany). Insulin was detected within 60 s by vector blue substrate kit (Vector Laboratory). Next, a rodent blocker (block endogenous mouse IgG, RBC and non-specific background, BIOCARE MEDICAL, Germany) was applied for 30 min and stained for the anti-BrdU antibody (monoclonal mouse anti-BrdU antibody, DAKO, Germany) in TRIS/BSA overnight at 4 °C. On the next day, a mouse on mouse HRP polymer (BioCare Medical, Germany) was administered on the sections for 45 min to develop brown colour with horseradish peroxidase (ImmPACT™ AMEC Red Substrate) at specifically stained positions on the slide. The colour changed to brown within 90 s. Images were taken with a light microscope (Leica microsystem, ICC50 HD) and evaluated with the ImageJ software.

For area calculation, one islet section was included upon containing at least five nuclei. From each mouse, four sections of 7 μm thickness were stained. Sections were selected for evaluation with a gap of four or five. A blinded examiner was provided with stained sections and an independent evaluation was conducted.

### Western blotting

The pancreas was crushed in liquid nitrogen with a mortar-pestle. Cells were subjected to the lysis buffer (Tissue PE LBTM, G-Biosciences) including a protease inhibitor (Protease ArrestTM, G-Biosciences). The lysate was incubated on ice for 10 min followed by centrifugation at 13,000 rpm for 30 min at 4 °C. The supernatant was collected and the protein level was measured by Bio-Rad Protein Assay. Thirty micrograrammes protein was denatured with 1X Roti-load (4X) and an equal amount was loaded in the gel. Afterwards, sodium dodecyl sulphate-polyacrylamide gel electrophoresis (SDS-PAGE) was performed to separate the protein followed by the transfer on activated polyvinylidene fluoride (PVDF; EMD) membrane with a semi-dry blotting chamber (Bio-Rad). The membrane was blocked with 5% milk powder at RT for 1 h and incubated with the respective primary antibodies (shown in Table 2) overnight at 4 °C. Next day, the membranes were washed with TBST and the secondary antibody (Polyclonal Goat Anti-Rabbit Immunoglobulins/HRP) was applied at RT for 1 h. Again, the membranes were washed with TBST and developed with an enhanced chemiluminescence system (ECL).

**Table 2:** Primary and secondary antibodies list

	Antibodies	Dilution	Company
**Primary antibody**	Rabbit beta-tubulin antibody, polyclonal	1:10,000	Abcam
**Primary antibody**	Rabbit AKT antibody, polyclonal	1:1000	Cell signaling
**Primary antibody**	Rabbit phospho-AKT (Ser473) antibody, polyclonal	1:1000	Cell signaling
**Primary antibody**	Rabbit anti-FoxO1 antibody, monoclonal	1:1000	Abcam
**Primary antibody**	Rabbit EGF antibody, polyclonal	1:500	Bioss
**Primary antibody**	Rabbit anti-FOXA2 antibody, monoclonal	1:1000	Abcam
**Primary antibody**	Rabbit anti PDX-1 antibody, polyclonal	1:500	Merck
**Secondary antibody**	Polyclonal Goat Anti-Rabbit Immunoglobulins/HRP	1:3000	Dako

### Tracking of hTERT-MSC by human Alu sequence/human DNA

Genomic DNA was isolated from the lung, liver, spleen, kidney, heart and pancreas from all three groups after 8 days. All tissues were incubated in 1.5 ml of lysis buffer (20 mM Tris-Cl, 5 mM EDTA, 400 mM NaCl and 1% SDS) containing 20 μl of Proteinase K (0.2 mg/ml) for 8 to 10 h at 56 °C followed by phenol/chloroform separation. Precipitation of the DNA was performed in ethanol. Samples were resuspended in RNase/DNase free water and the DNA concentration was quantified by using a NanoDrop spectrophotometer (NanoDrop, USA) and 1 μg DNA was used for PCR. For the analysis, 0.5 × 10^6^ hTERT-MSC were considered (similar number of cells used for transplantation) and the standard curve was constructed with tenfold serial dilution. PCR was performed with an initial denaturation at 95 °C for 10 min, followed by 40 cycles of denaturation (95 °C, 15 s), annealing (60 °C, 30 min) and extension (60 °C, 1 min) and followed by melting curve. Quantification of hTERT-MSC number was performed with Ct value through the standard curve.

### Statistical analysis

Statistical analysis was performed with Prism 8 (GraphPad, USA) using the unpaired and paired *t* test or the one- and two-way ANOVA, as appropriate. Data represent the mean ± standard error (SEM) unless otherwise stated. A *p* value of < 0.05 was considered significant.

## Results

### MSC allowed faster recovery from abdominal surgery

According to German animal welfare guidelines, the animals’ health appearance was evaluated every day based on a scoring system designed for abdominal surgery in mice (supplement 1). A score of two or more was considered to call for treatment, while score zero was associated with well-being. As compared to control, mice from the IPR group showed a significantly lower score on the fourth day after pancreatic surgery. At the end of the experiment, the IPR group (*p* < 0.001) had completely recovered from the pancreatic injury, whereas a residual impairment was apparent in both the IVR and the control group (Fig. 1b). Survival curves demonstrated a non-significant benefit of the transplanted groups (Fig. 1c). Eight days after partial pancreatectomy, a lower mean blood glucose level was observed in the IVR group (12.7 ± 0.74 mmol/l) and IPR group (11.5 ± 0.63 mmol/l) but without significance compared to control mice (13.6 ± 0.73 mmol/l) as shown in Fig. 1d. The body weight of the mice was measured daily. While no statistical difference was observed between groups in the short time span of 8 days (control 33.56 ± 1.25 g, IVR 34.83 ± 1.35 g; IPR 34.85 ± 1.16 g), the animals recovered weight faster after pancreatic surgery in the transplanted groups (Fig. 1e).

**Fig. 2:**
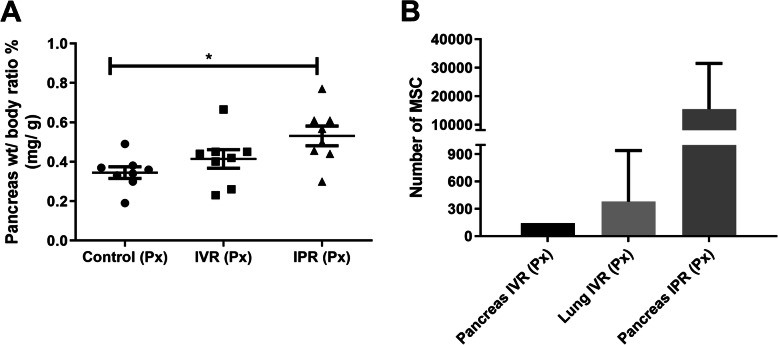
Pancreatic weight and the localization of hTERT-MSC following partial pancreatectomy. **a** Demonstrates the pancreatic weight/body weight ratio in percentage in box plot with each dot representing individual mouse, *n* = 8. **b** Localization of hTERT-MSC by human Alu sequence. 0.5 × 10^6^ hTERT-MSC were transplanted via IVR or IPR and the control group with the same volume of MEM intravenously post pancreatectomy (Px). After 8 days, mice were sacrificed and investigated for human Alu sequence. The representative figure shows the number of hTERT-MSC obtained after 8 days of pancreatectomy. After intravenous transplantation (IVR) of hTERT-MSC, majority of MSC were exposed to lung microvasculature and detected in four out of eight lungs. However, few cells homed to the damaged pancreas, only one pancreas out of eight was positive. hTERT-MSC DNA was not detected in other retrieved organs such as the liver, spleen, kidney, heart and in the control group. In contrast, IPR displayed the presence of a significantly higher number of hTERT-MSC. Further, hTERT-MSC DNA remained undetected in undamaged organs. Intravenous route (IVR), intrapancreatic route (IPR) and human telomerase reverse transcriptase mesenchymal stem cells (hTERT-MSC). Data are given as mean ± SEM, *p < 0.05

As part of the routine screening, the lung, spleen, kidney and heart wet weights were monitored without finding discrepancies between the experimental groups (data not shown). However, a significant difference in pancreatic weight/body weight ratio in percentage (mg/g) was observed. The mean wet weight ratio of the residual pancreas was increased in the IPR group compared to control (0.5314 ± 0.601% versus 0.3143 ± 0.024, *p* < 0.05), but not to the IVR group (0.3829 ± 0.035) as demonstrated in Fig. 2a.

### Engraftment of hTERT-MSC

After 8 days of Px, we assessed the distribution of the infused hTERT-MSC with the human Alu sequence in mouse genomic DNA of the lung, liver, spleen, kidney, heart and pancreas with the PCR. The presence of hTERT-MSC DNA was detected based on the Ct value. In the IVR group, most of the cells were trapped in the lungs, but few would reach the damaged pancreas. An average of 380 ± 280 were present in the lungs while pancreas showed 142 ± 1 of hTERT-MSC in the IVR group (Fig. 2b). Human DNA was undetected in the liver, spleen, kidney and heart of the IVR group. In the IPR group, the human Alu sequence was observed in the pancreas with an average of 15,397 ± 7207 hTERT-MSC and remained undetected in other organs (Fig. 2b). In the control group, no sign of the human Alu sequence was observed.

### Proliferation of pancreatic β-cells was supported by MSC

**Fig. 3:**
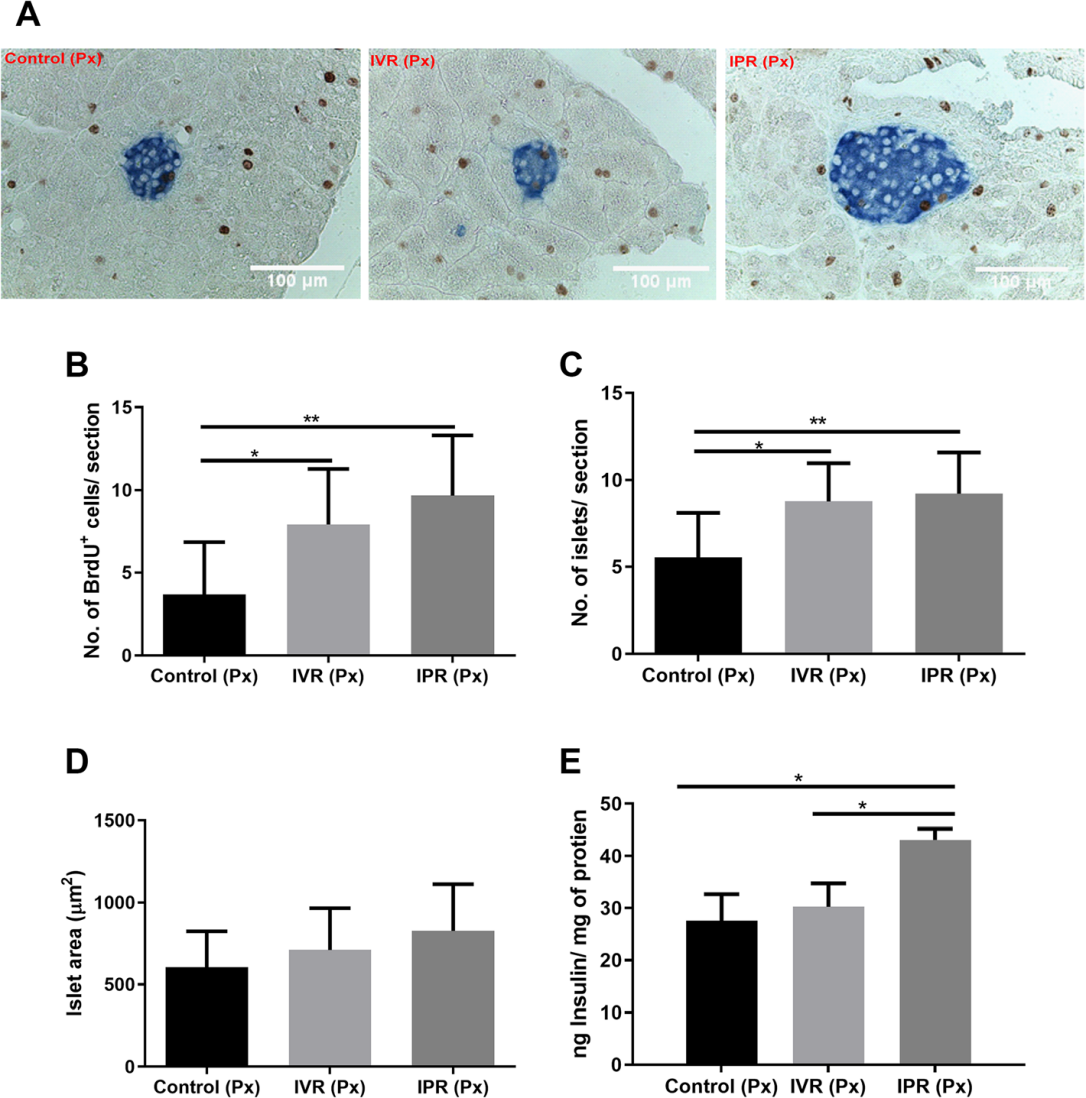
hTERT-MSC increased pancreatic β-cell proliferation. Control underwent pancreatectomy with intravenous injection of culture medium. **a** Representative pancreatic sections stained with anti-insulin (blue) and anti-BrdU antibodies. A brown spot within the islets surrounded by blue-coloured cytoplasm represented the β-cell nucleus labelled with BrdU. **b** Total number of BrdU^+^ cells within the sectioned islet area. **c** Number of islets per field (two or three). **d** Islets area and **e** insulin content in the residual pancreas after 8 days of pancreatectomy. Data represent mean ± SEM. From each mouse, at least four sections with 7 μm thickness were picked with an interval of five subsequent sections. One-way ANOVA with Tukey’s post hoc test was used for analyzing the data. Insulin area was calculated with the Image J software. From each group, intravenous route (IVR), intrapancreatic route (IPR) of transplanted human telomerase reverse transcriptase mesenchymal stem cells (hTERT-MSC), 32 sections per group were analyzed to measure BrdU positive cells and islet areas, *n* = 7, *p < 0.05, **p < 0.01

To shed light on the underlying effects of MSC on the injured residual pancreas, cellular nuclei preparing for division by chromatin remodelling were labelled with bromodeoxyuridine (brown colour) within the islets (blue colour) as shown in Fig. 3a. In the IPR group (9.6 ± 0.85), the count of proliferating pancreatic β-cells was significantly increased in comparison to control (5.0 ± 1.16; *p* < 0.0044; Fig. 3b). Control and IVR (*p* < 0.024) also displayed a statistical difference, but this effect was diluted between IVR and IPR (*p* < 0.4205). Further, the number of islet sections per field was calculated. The IPR group had a higher number of islets per field (9.2 ± 0.55) than control (5.53 ± 0.71; *p* < 0.0034) but displayed non-significant statistics compared with IVR (8.7 ± 0.61; *p* < 0.86) as shown in Fig. 3c. Moreover, the mean islet area was enhanced, without achieving significance (IPR 826 ± 76.30 μm^2^, IVR 711 ± 68.12 μm^2^, and control 604 ± 58.57 μm^2^; Fig. 3d).

### MSC induced the transcription of growth factors and attenuated local inflammation

**Fig. 4:**
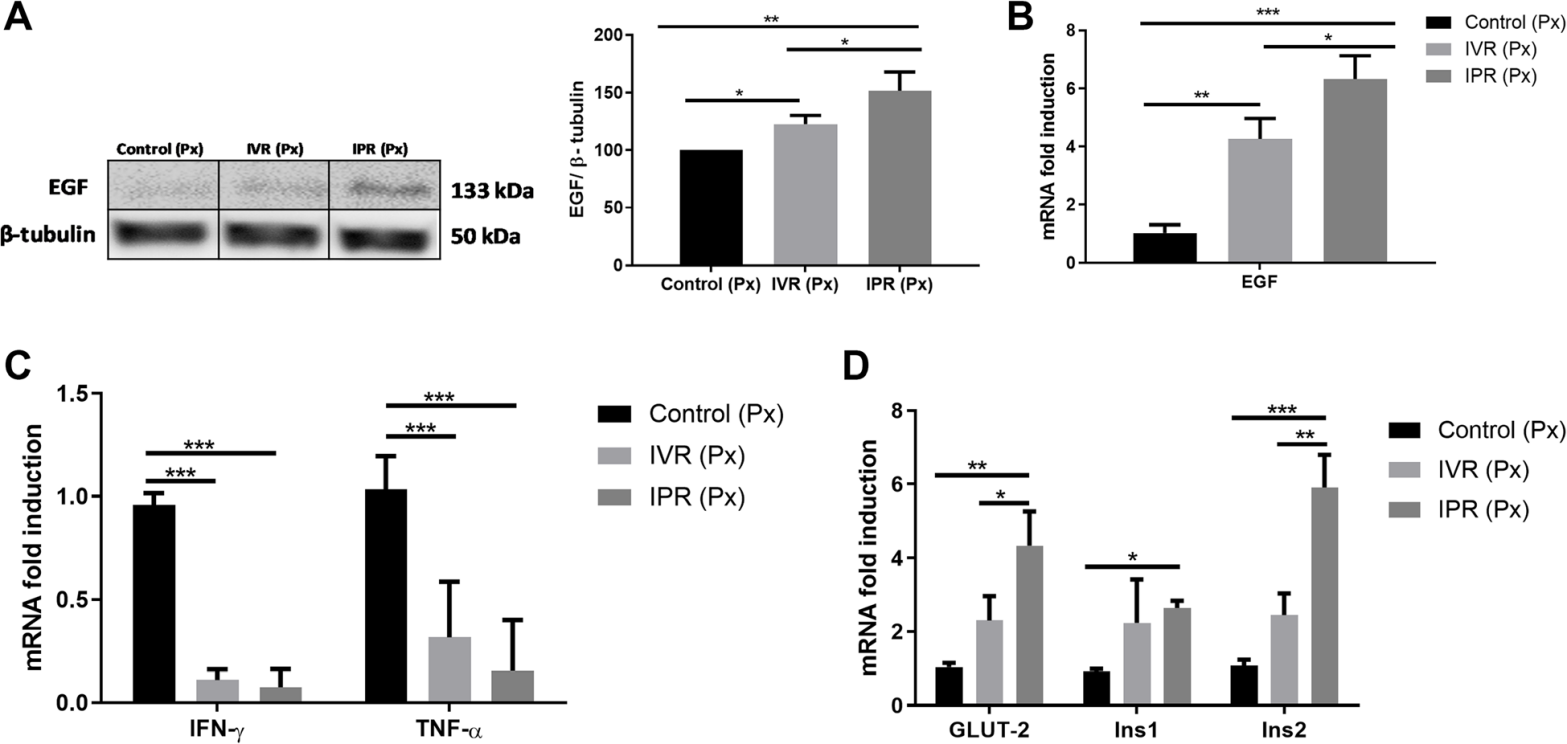
hTERT-MSC stimulated growth factors and anti-inflammatory markers in the residual pancreas. **a** EGF protein was evaluated 8 days after pancreatectomy and MSC administration. Route dependent increase in EGF protein was analyzed by Western blot, *n* = 3. Panel **b** demonstrates that EGF transcripts were accordingly increased, *n* = 4. **c** Expression of IFN-γ and TNF-α genes in the residual pancreas. MSC transplantation reduced IFN-γ and TNF-α transcript levels in both IVR and IPR groups as compared to control, *n* = 4. **d** Gene expressions of GLUT-2 and *Ins2* were enhanced in IPR-injected mice compared to controls and IVR-transplanted mice. *Ins1* gene expression increased in IPR, but not in IVR-treated mice compared to control, *n* = 4. Epidermal growth factor (EGF), glucose transporter 2 (GLUT-2), preproinsulin 1 (*Ins1*), preproinsulin 2 (*Ins2*), *interferon gamma* (IFN-γ), tumour necrosis factor alpha (TNF-α), intravenous route (IVR), intrapancreatic route (IPR) and human telomerase reverse transcriptase mesenchymal stem cells (hTERT-MSC). Data are given as mean ± SEM, *p < 0.05, **p < 0.01, ***p < 0.001, post hoc test versus sham-operated control group

As we have observed the pro-proliferative effects of the hTERT-MSC administration, we analyzed epidermal growth factor (EGF), known to facilitate recovery from chemically-induced diabetes in mice [34, 35]. Surprisingly, EGF variation was route-dependent after hTERT-MSC administration. EGF protein expression was highest in the pancreas of the IPR group as opposed to control (*p* < 0.002) and IVR (*p* < 0.03) (Fig. 4a). This finding was confirmed with EGF mRNA transcripts in IVR (*p* < 0.0092) and IPR (*p* < 0.001) compared to control (Fig. 4b).

It is acknowledged that surgical trauma leads to a local increase of inflammatory cytokines [36]. Interestingly, hTERT-MSC infusion attenuated the transcription of IFN-γ and TNF-α at the surgical site. Relative transcription of IFN-γ was statistically reduced in both the IPR (*p* < 0.001) and IVR group (*p* < 0.001) as opposed to control, whereas no difference was observed between the IPR and IVR groups (Fig. 4c). Similarly, TNF-α mRNA transcription followed the same pattern. Subsequent to transplanting hTERT-MSC, the mRNA level of TNF-α decreased in the IPR (*p* < 0.0006) and IVR groups (*p* < 0.0009) compared to Px with sham transplantation (Fig. 4c).

### Endocrine function after hemipancreatectomy was rescued by MSC

Next, we analyzed whether the observed effects of MSC also had an impact on the endocrine function of the residual pancreas. The increment of GLUT-2 expression was reported to induce synthesis of insulin [37]. Correspondingly, the GLUT-2 transcription in the IPR group achieved significance as compared to control (*p* < 0.01) and IVR (*p* < 0.05, Fig. 4d) after transplantation of hTERT-MSC. Enhancement of pancreatic insulin synthesis was further confirmed by analyzing *Ins1* and *Ins2* transcripts. *Ins2* expression was augmented after both local (*p* < 0.0002) and systemic injection (*p* < 0.004) in comparison with control. Likewise, *Ins1* mRNA expression also displayed a statistical difference among control and IPR group (*p* < 0.02; Fig. 4d). Concomitantly, as shown in Fig. 3e, the total pancreatic insulin content was significantly increased in the IPR group (43.60 ± 1.22 ng insulin/mg protein) as opposed to the control group (27.60 ± 2.92 ng insulin/mg protein; *p* < 0.0140) and IVR group (30.28 ± 2.57 ng insulin/mg protein; *p* < 0.023).

### MSC initiated the expression of transcription factors associated with endocrine pancreatic progenitor cells

**Fig. 5:**
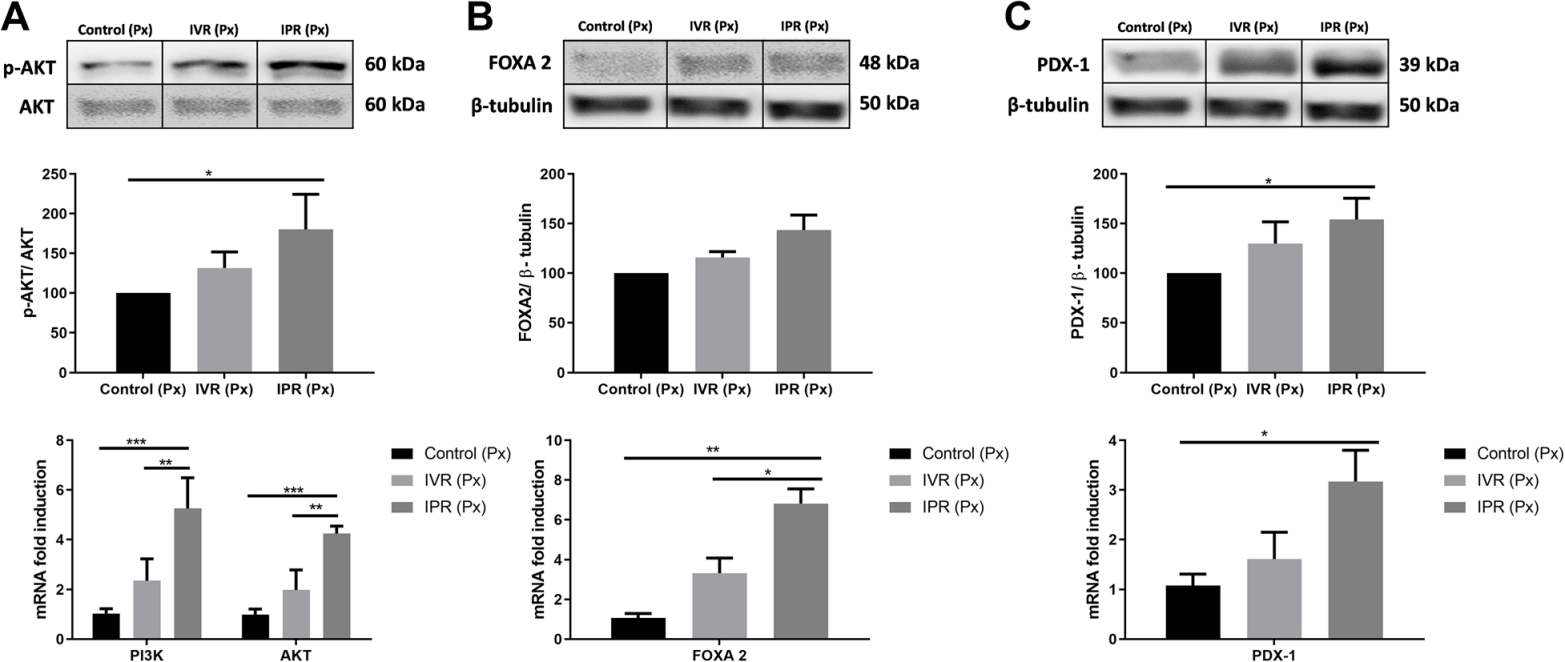
hTERT-MSC activated factors specific for endocrine pancreatic progenitor cells. In each of the panels **a**–**c**, the two upper images represent the expression of the protein and the lower image fold expression of gene normalized to the control (Px only group). **a** Represents the increase of phosphorylated AKT protein expression in MSC-infused pancreas (upper and middle panels) and concomitant augmentation of the quantity of PI3K and AKT transcripts (lower panel). Significant differences were observed between IPR and control (*p* < 0.03) at the protein level and all experimental groups at the mRNA level. **b** Demonstrates the enhancement of forkhead box protein A2 (*FOXA2*) concentration in IVR and IPR MSC-treated groups. **c** Pancreatic and duodenal homeobox 1 (PDX-1) showed a significant augmentation between control and IPR by Western blot (*p* < 0.02) and RT-PCR (*p* < 0.017). Intravenous route (IVR), intrapancreatic route (IPR), phosphatidylinositol-4,5-bisphosphate 3-kinase (PI3K), protein kinase B (AKT), human telomerase reverse transcriptase mesenchymal stem cells (hTERT-MSC) and beta-tubulin band are similar in Figs. 4 and 5 (same membrane). Data are given as mean ± SEM. *n* = 3, *p < 0.05, **p < 0.01, ***p < 0.001, comparisons performed with the control

We aimed to gain insight into the mechanisms behind the observed convalescence of the pancreatic insulin production. Since MSC are considered to induce the proliferation of pancreatic β-cells and the AKT/FOXA2/PDX-1 signalling pathway is seen as a potential mediator of this process, AKT protein was measured by Western blot and RT-PCR in partially pancreatectomized mice after hTERT-MSC infusion. The phosphorylation of AKT was indeed increased in the residual pancreas of IPR as opposed to control (*p* < 0.03, upper and central panels of Fig. 5a). Relative transcription of both PI3K and AKT was significantly elevated in the IPR group compared with control (AKT; *p* < 0.001, PI3K; *p* < 0.001) and IVR (AKT; *p* < 0.01, PI3K, *p* < 0.01), respectively (lower panel of Fig. 5a). Furthermore, after 8 days, when the pancreatic β-cell proliferation was supposed to be at maximum [38], FOXA2 protein expression remained insignificant but higher gene expression was observed in the IPR group as compared to the control (*p* < 0.009) and IVR (*p* < 0.04, Fig. 5b). Moreover, PDX-1 was reported to increase along with pancreatic β-cell regeneration [39, 40]. Therefore, PDX-1 was measured in addition. Again, a significant difference was monitored with hTERT-MSC administered locally (IPR group) at both the protein (*p* < 0.02, Fig. 5c; upper and central) and mRNA level (*p* < 0.017, Fig. 5c; lower) when compared to control. No difference could be detected between the IVR group and control.

### MSC downregulated FoxO1 expression

**Fig. 6:**
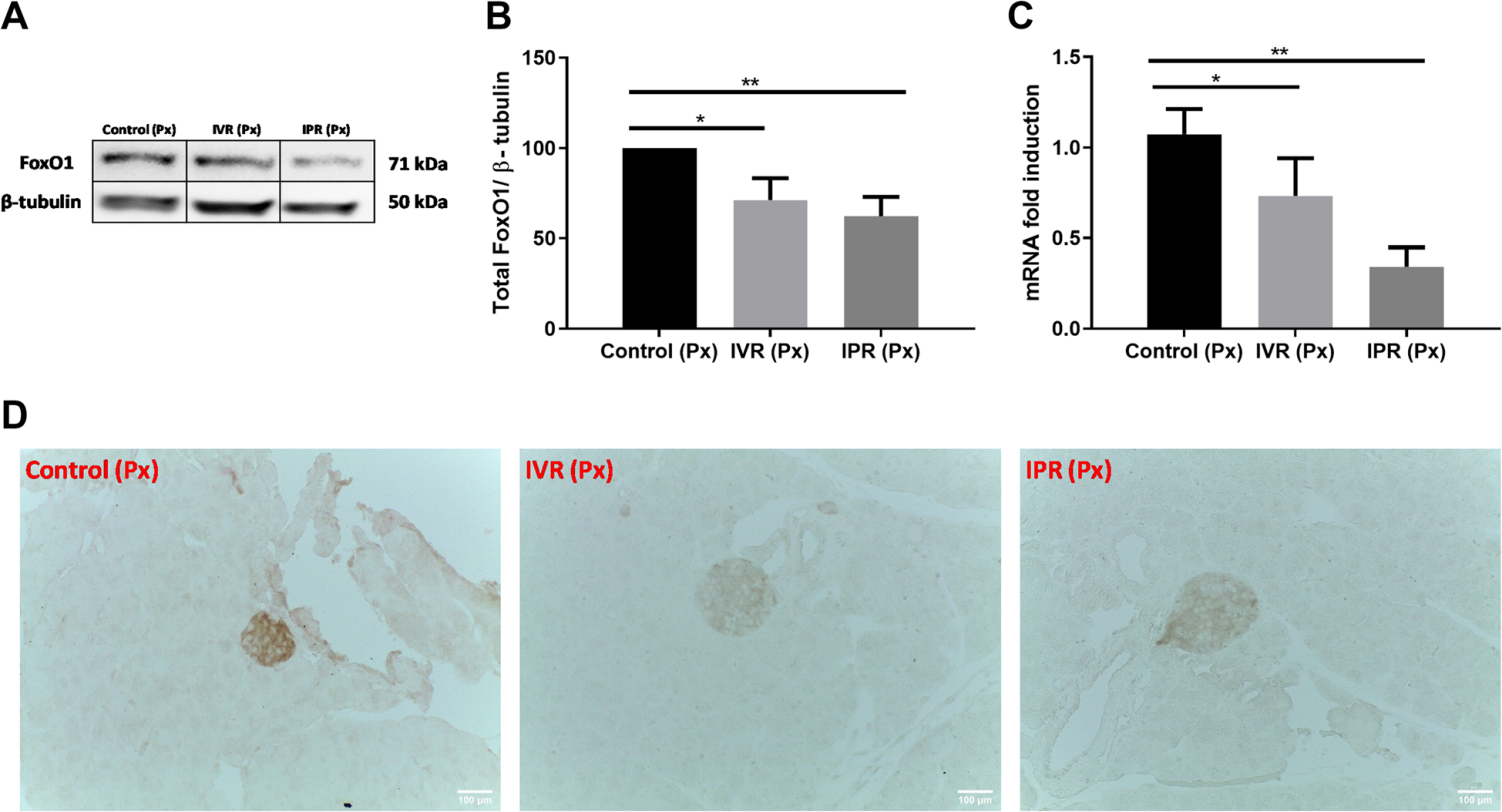
hTERT-MSC downregulated FoxO1 to regenerate β-cells. **a** After 8 days of MSC infusion in partially pancreatectomized mice, MSC route-dependent downregulation of forkhead box protein 1 (FoxO1) protein was monitored. FoxO1 expression was significantly reduced in IPR and IVR compared to control on protein (**b**) and gene (**c**) level. **d** Immunohistochemistry was performed to investigate FoxO1 straining within the pancreatic islets and representative sections are shown. Compared with control FoxO1 staining was decreased in IVR and IPR group. Intravenous route (IVR), intrapancreatic route (IPR) and human telomerase reverse transcriptase mesenchymal stem cells (hTERT-MSC). Data represent the mean ± SEM. *n* = 3, *p < 0.05, **p < 0.01, ***p < 0.001, statistical comparisons were performed with the control

Another crucial transcription factor to consider in respect of potential β-cell fates is forkhead box protein 1 (FoxO1). It can have a major impact on initiating the proliferation of pancreatic β-cells and activating pancreatic progenitor markers [17]. In regenerative and highly proliferative microenvironments, phosphorylation of AKT was noticed to downregulate the expression of FoxO1 [41], which in turn is downregulated by PDX-1 through the FOXA2 switch for β-cell regeneration [42]. In the present experiment, FoxO1 protein was indeed significantly downregulated (*p* < 0.01) in the IPR group compared with the control (Fig. 6a). Similarly, a reduction in the mRNA expression of the FoxO1 gene in the IVR group (*p* < 0.028) was observed and mRNA was further decreased in the IPR group (*p* < 0.002, Fig. 6b). Within the islets, a moderate reduction of FoxO1 in the IVR and IPR group was observed as shown in Fig. 6c.

## Discussion

The purpose of this study was to dissect the effect of human MSC injected either in a systemic or local fashion on the regeneration of pancreatic β-cells. Hemipancreatectomy in rodents is a model of pancreas regeneration but has not been investigated in the context of human MSC. Therefore, this is the first study to demonstrate the beneficiary effect of human hTERT-MSC in β-cell regeneration subsequent to hemipancreatectomy.

MSC were reported to reverse hyperglycaemia in type 1 diabetic rodent models as they enhanced insulin secretion and modified the immunological niche within pancreatic tissue [10, 43–47]. In these studies, the intravenous delivery of stem cells was performed in rodents and patients [48, 49]. However, this approach leads to a vast loss of MSC due to pulmonary entrapment [50, 51], which can be counteracted by local transplantation. Therefore, we compared the therapeutic outcome of the systemic (intravenous route; IVR) and local administration (intrapancreatic route; IPR) of MSC. Of note, an additional control group where 100 μl MEM was injected directly into the pancreas post-Px to avoid self-regeneration was also considered (data not shown) but we did not find any difference between the controls.

Interestingly, pancreatic weight upon body weight ratio and scoring system designed for abdominal surgery reflected a beneficial effect on the convalescence of the damaged pancreas by hTERT-MSC. An operative insult to the pancreas does not simply include endocrine function but has a significant impact on the whole organ including exocrine epithelial cells and stromal tissue. Hence, it could be a reason for not achieving significance in the blood glucose level among different groups in this study. Moreover, in this study, a single low dose of 0.5 × 10^6^ hTERT-MSC was investigated. Further, multiple doses or single dose with higher number of MSC would provide a better insight for the initiation of a clinical trial, as MSC transplantation in IPR remains unexplored as opposed to IVR in human pancreatic injury.

The functional effects of transplanted MSC depend upon migration towards damaged tissue, including pancreatic islets [52]. The detection of human Alu sequence allowed to identify human DNA of transplanted cells in rodents [53, 54]. Eight days after injection of MSC, human DNA was observed in injured tissue (approximately 63%; IPR) and not detected in non-operative organs (liver, spleen, kidney, and heart), suggesting the assumption of an active locomotor response towards the inflamed site.Growth factors such as EGF, known to stimulate the proliferation of pancreatic β-cells, are potential mediator of the observed beneficial effects of MSC. EGF could be either provided in a paracrine manner directly by hTERT-MSC or by other cells upon stimulation by MSC. EGF was adopted in combination with ciliary neurotrophic factor (CNTF) which maintained β-cell mass from pre-existing β-cells [35, 55]. Furthermore, EGF was shown to restore the amount of the functional β-cell mass in rats, thereby attenuating hyperglycaemia in diabetic mice [19, 56]. Some studies imply that EGF was produced by the ductal or acinar cells, but recent lineage tracking experiments contradicted this conclusion [34, 35, 57–60]. In our study, an enhanced murine EGF expression was observed in the untreated residual pancreas, indicating that EGF was produced by pancreatic cells rather than the MSC. The EGF action on β-cells could support the GLUT-2 transcription. In a transgenic mouse model featuring a mutated EGF-receptor (EGF-R) within the PDX-1 promotor resulted in a decreased GLUT-2 gene expression [61]. Indeed, in this experiment, the amount of GLUT-2 in the IPR group was higher, enhancing the β-cells glucose-stimulated insulin response. Furthermore, the transcription of both the *Ins1* and *Ins2* gene was significantly elevated in the IPR group as opposed to control and IVR groups. In consequence, the pancreatic insulin content in the IPR-treated mice was also markedly higher than in the other groups.

Moreover, MSC possess immunomodulatory properties by releasing specific cytokines at the site of nerve, pancreatic islet and renal injury in diabetic mice [47, 62, 63]. Ezquer et al. reported a systemic and local reduction in the abundance of auto-aggressive T cells in favour of regulatory T cells in a murine model of low-dose streptozotocin-induced diabetes treated with autologous MSC [46]. In a setup of partial pancreatectomy, hTERT-MSC administration downregulated the local IFN-γ and TNF-α gene expression. Interestingly, both regional (IPR) and systemic (IVR) routes delivered a therapeutic effect, indicating that cells trapped in the lungs in the IVR group might secrete anti-inflammatory molecules and trophic factors as well [64].

In a similar manner, the expression of the pancreatic progenitor transcription factors FOXA2 and PDX-1 was reported to be enhanced following Px, which augmented the proliferation and regeneration of β-cells from pre-existing ones [17, 65–68]. Therefore, we further evaluated the effect of administered hTERT-MSC on the residual regenerative pancreas. FOXA2 is an early definitive endoderm marker and serves as an upstream modulator of PDX-1 [69]. We confirmed an increased expression of both FOXA2 and PDX-1 subsequent to hTERT-MSC administration.

To further investigate the underlying molecular mechanism responsible for the observed pancreatic β-cell regeneration, we also explored the PI3K/AKT, ERK and TGF-β pathways. Liu et al. recently suggested that hTERT-MSC activates AKT and ERK1/2 signalling in cultivated rat insulinoma-derived INS-1E β-cells [70], which was now confirmed with our data in vivo. Furthermore, the resection of pancreatic tissue was reported to facilitate IRS2-AKT signalling in the residual pancreatic cells, resulting in pancreatic β-cell proliferation via FoxO1 regulation [17]. However, treatment with hTERT-MSC did not further increase the IRS2 expression at the transcription level in our experiment (data not shown). Likewise, the expression of ERK and TGF-β were higher after Px when compared to the native pancreas, but independent of hTERT-MSC administration (data not shown).

Further, we analyzed the FoxO1, considered an effective regulator of pancreatic β-cell growth and differentiation, i.e. by suppression of PDX-1 [71–73]. According to our data, the expression of FoxO1 was reduced in the transplanted groups, corresponding to the elevated expression of FOXA2 and PDX-1.

## Conclusion

Taken together, we provide evidence that MSC administration through both IVR and IPR increased the proliferation of autochthonous pancreatic β-cells. However, moderate MSC beneficiary effect (insulin reserve and surgical health assessment) of IPR over IVR was observed. Therefore, our data encourage the execution of MSC clinical trials with IPR in patients with distal pancreatectomy.

## Supplementary Information

**Additional file 1.**

## Data Availability

All relevant data and material to reproduce the findings are available in the manuscript.

## Abbreviations

AKT: Protein kinase B
β-cell: Beta-cell
BrdU: Bromodeoxyuridine
EGF: Epidermal growth factor
ELISA: Enzyme-linked immunosorbent assay
FoxO1: Forkhead box protein 1
*FOXA2*: Forkhead box protein A2
GLUT-2: Glucose transporter 2
hTERT-MSC: Human telomerase reverse transcriptase mesenchymal stem cells
IFN-γ: Interferon gamma
IHC: Immunohistochemistry
*Ins1*: Preproinsulin 1
*Ins2*: Preproinsulin 2
IPR: Intrapancreatic route
IVR: Intravenous route
MSC: Mesenchymal stem cells
PDX-1: Pancreatic and duodenal homeobox 1
PI3K: Phosphatidylinositol-4,5-bisphosphate 3-kinase
Px: Partial pancreatectomy
RPL32: Ribosomal Protein L32
RT-PCR: Real-time polymerase chain reaction
TNF-α: Tumour necrosis factor alpha
