# Supplementary material for: Mesenchymal stem cells promote pancreatic β-cell regeneration through downregulation of FoxO1 pathway
Source: Stem Cell Res Ther. 2020 Nov 25;11:497. doi: 10.1186/s13287-020-02007-9 (PMC7687794; doi:10.1186/s13287-020-02007-9)
Supplement: Supplementary file 1 — Additional file 1. [file 13287_2020_2007_MOESM1_ESM.pdf]

Score sheet for the regeneration experiment.

Mouse ID:	Date:								
	Time:								
**1. Body Weight**	Score								
Based on initial weight []									
Based on control group weight []									
Uninfluenced or rise	0								
Reduction, but <10%	1								
Reduction > 10%	2								
Reduction > 20%	3								
**2. General condition**									
Shiny eyes, body openings and skin clean	0								
Cloudy eyes, increased muscle tone, more visible breathing	1								
Eyes sunken dull, sticky body openings, increased breathing	2								
Abnormal posture, the animal feels cold, eyes closed, cramps, paralysis, breath sounds, bluish mucous membranes, diarrhea	3								
**3. Spontaneous behavior**									
Attentive, curious, straightening, quick movements	0								
Decreased reactions, movement reduced, restricted or excessive activity	1								
Partial separation from the group, movement reduced, pain when walking	2								
Apathetic, no reaction or aggressiveness in handling, severely restricted movement, isolation, drags forward.	3								
**5. Other termination criteria**									
Self-injury (e.g. in case of excessive itching)	3								
**Total score**									
